# Estimating the number of livebirths to Hepatitis C seropositive women in England in 2013 and 2018 using Bayesian modelling

**DOI:** 10.1371/journal.pone.0274389

**Published:** 2022-11-21

**Authors:** Emily Dema, Julian Stander, Mario Cortina-Borja, Claire Thorne, Heather Bailey

**Affiliations:** 1 Institute for Global Health, University College London, London, United Kingdom; 2 Centre for Mathematical Sciences, School of Engineering, Computing and Mathematics, University of Plymouth, Plymouth, United Kingdom; 3 Population, Policy and Practice Research and Teaching Department, Great Ormond Street Institute of Child Health, University College London, London, United Kingdom; Public Library of Science, UNITED KINGDOM

## Abstract

**Background:**

The UK National Screening Committee currently recommends against antenatal screening for Hepatitis C virus (HCV) infection in England due to lack of HCV prevalence data and treatment licensed for use in pregnancy. We aimed to produce regional and national estimates of the number and proportion of livebirths to HCV seropositive women in England in 2013 and 2018.

**Methods:**

A logistic regression model fitted in the Bayesian framework estimated probabilities of HCV seropositivity among 24,599 mothers delivering in the North Thames area of England in 2012 adjusted by maternal age and region of birth. These probabilities were applied to the underlying population structures of women delivering livebirths in England in 2013 and 2018 to estimate the number of livebirths to HCV seropositive women in these years nationally and by region. The Bayesian approach allowed the uncertainty associated with all estimates to be properly quantified.

**Results:**

Nationally, the estimated number of livebirths to women seropositive for HCV for England was 464 (95% credible interval [CI] 300–692) in 2013 and 481 (95%CI 310–716) in 2018, or 70.0 (95%CI 45.0–104.1) per 100,000 and 76.9 (95%CI 49.5–114.4) per 100,000 in these years respectively. Regions with the highest estimated number of livebirths to HCV seropositive women in 2013 and 2018 included London with 118.5 and 124.4 and the South East with 67.0 and 74.0 per 100,000 livebirths.

**Conclusion:**

Few previous studies have investigated HCV among pregnant women in England. These findings complement and supplement existing research by providing national and regional estimates for the number of livebirths to HCV seropositive women in England. Bayesian modelling allows future national and regional estimates to be produced and the associated uncertainty to be properly quantified.

## Introduction

Worldwide, around 71 million people were estimated to be living with chronic hepatitis C virus (HCV) infection in 2015 [1] while updated estimates for 2019 indicated 14.9 million viraemic HCV infections in women aged 15–49 years [2]. Injecting drug use (IDU) is the most important risk factor for HCV in Europe, whereas HCV epidemics in high-prevalence regions in parts of Asia and Africa have been driven by unsafe medical procedures [3, 4]. Treatment with direct-acting antivirals (DAAs) can provide a virologic cure of all major subtypes of HCV within 8–12 weeks for most people [5], and in the UK their roll-out has resulted in recent falls in the number of people living with chronic HCV infection (from an estimated 174,000 in 2015 to 118,000 in 2019) [6]. However, worldwide a large majority of HCV infections remain undiagnosed, highlighting the need for effective surveillance and control policies if the global goal of HCV elimination by 2030 is to be achieved [4, 7].

Most children with HCV acquire it vertically from their mothers. A 2014 meta-analysis including 109 studies reported a 5.8% (95% confidence interval [ci] 4.2%–7.8%) vertical HCV transmission rate, with this increasing to 10.8% (95%ci 7.6%–15.2%) among women with HIV/HCV co-infection [8]. Pregnant women remain a neglected group for HCV surveillance in England due to lack of an antenatal screening programme. In 2018, the UK National Screening Committee (NSC) advised against routine screening for HCV among pregnant women in the UK, citing lack of a national estimate for HCV seroprevalence among UK pregnant women, as well as the absence of evidence regarding the effectiveness of treatment with DAAs among pregnant women and young children [9]. Since then, a small Phase I clinical trial demonstrated safety and efficacy of Ledipasvir / Sofosbuvir in pregnant women [10] and DAAs have been licensed for children ≥3 years [11]; postnatal treatment with DAAs for women diagnosed with HCV in pregnancy could be another approach, although postpartum loss to follow-up has been identified as an important issue in several studies [12–15]. In 2020, both the American Association for the Study of Liver Diseases (AASLD) and the European Association for the Study of Liver (EASL) updated their guidelines to recommend HCV screening for all pregnant women [16, 17]. The UK NSC recommendations regarding HCV screening in pregnancy are due for review in 2021/22 but currently screening for HCV in pregnancy in the UK remains risk-based, for example for those women reporting a history of injecting drug use. A recent global policy review found that only 26% of national strategic plans and/or clinical practice guidelines included recommendations on HCV screening in pregnancy [18]. However, universal screening of pregnant women for HCV infection could facilitate earlier diagnosis of infection in children, as well as identify women for treatment with DAAs, contributing to goals of HCV elimination in England and globally [3, 19, 20].

Very few studies have assessed HCV seroprevalence among pregnant women in England, with only three published in the past 20 years, two of which were based on surveillance data from the North Thames region [21–23]. One study used convenience sampling of women in antenatal clinics, which potentially excludes the group of women that do not routinely attend antenatal clinics [23].

The North Thames studies provide insight into HCV seroprevalence in an unselected population delivering liveborn infants, most recently in 2012, with a significant decrease in seroprevalence rates over time from 190 per 100,000 pregnant women in 1998 to 95 per 100,000 in 2012 [21, 22]. However, the North Thames region is unrepresentative of England as a whole in terms of maternal age and country of birth [24, 25], both key factors associated with HCV seropositivity. For example, 2012 data showed the highest HCV seropositive rate of 0.366% among young women born in Eastern Europe, with this peaking at age around 27 years, while HCV seroprevalence rates for women born in Southern Asia and the UK were 0.162% and 0.019% respectively and increased with age [22]. The difference in maternal sociodemographic characteristics between North Thames and other regions of England limits generalisability of these previous findings on antenatal HCV seroprevalence. Therefore, in order to address the absence of national data, we applied the North Thames 2012 estimates of HCV seroprevalence specific to maternal age group and region of origin [22] to data on number of livebirths in these maternal demographic groups nationally in 2013 and 2018, in order to estimate the number of livebirths to HCV seropositive women in England and its regions in these years.

## Material and methods

### North Thames 2012 dried blood spot data

The North Thames neonatal screening laboratory covers a region including North London, Bedfordshire, Hertfordshire and Essex (i.e. inner and outer London, as well as mixed urban and rural districts). In 2012, around half of deliveries in the North Thames area were to women born outside of the UK (19% in the Asia-Pacific region, 14% elsewhere in Europe, 10% in Africa) and North Thames overall accounted for around a sixth of all livebirths in England [22]; similar proportions applied in 2018.

Dried blood spot (DBS) samples (Guthrie cards) are routinely collected from infants for metabolic screening at about 1 week of age using a heel prick [26]. As part of the previous study, residual samples of neonatal DBS received at the North Thames neonatal screening laboratory during one quarter in 2012 (1 April to 30 June 2012) were tested for HCV antibodies, after exclusion of multiple births [22]. The presence of HCV antibodies in the neonatal sample reflects maternal antibody status, due to passive transfer to the fetus *in utero* [27]. Residual DBS samples were matched to birth registration records to obtain parental country of birth and maternal age before being irreversibly anonymised and only then were they tested for HCV antibodies, using unlinked anonymous surveillance methods as previously described [22]. Of the 31,467 samples included in the previous study, 30 (0.095%) were HCV seropositive.

In this paper we analyse data from a subset of *n* = 24,599 samples from the North Thames 2012 survey [22] with information available on maternal age and region of birth as well as maternal HCV serostatus. The proportion of samples with HCV antibodies was similar for those included in these analyses (25 of 24,599) vs those excluded due to missing data on maternal age or region of birth (5 of 6,868 samples).

Ethics approval for the original study was granted by the East Midlands Research Ethics Committee (reference 12/EM/0488). Further ethics approval was not required for use of the anonymised dataset in this statistical modelling study.

### ONS live births data

Data for livebirths in England occurring in 2013 and 2018 were obtained from the Office for National Statistics (ONS) Nomis database, which provides regional-level information on livebirths by maternal age and maternal region of birth (RoB) [24, 25]. Maternal RoB was classified as UK, Rest of EU (with EU defined at time of data collection), and Other regions (including other European, Asia-Pacific, African and the American countries).

### Statistical analysis

Firstly, a logistic regression model was fitted in the Bayesian framework to estimate probabilities of HCV seropositivity adjusted by maternal age and RoB, among the 24,599 samples in the North Thames 2012 dataset. Maternal age and RoB have been associated with HCV seropositivity in previous seroprevalence studies [21, 22]. Maternal age was included in the model as a grouped covariate in order to match the data available from the ONS’s Nomis database. The outcome of interest was HCV serostatus.

Secondly, the probability estimates yielded by our modelling were applied to the underlying population structure of women delivering livebirths nationally in 2013 and 2018 (ONS data) to estimate the number of livebirths to HCV seropositive women in these years nationally and by region in England. The Bayesian approach allows us to quantify properly the uncertainty associated with our estimates.

### Bayesian analysis

We fitted the logistic regression model:

$$y_{ij}∼\mathrm{Bin}(n_{ij},p_{ij})$$

$$\log\left(\frac{p_{ij}}{1-p_{ij}}\right)=\beta_{0}+\beta_{1}I(i\;\mathrm{is}\;25‐29)+\beta_{2}I(i\;\mathrm{is}\;30‐34)+\beta_{3}I(i\;\mathrm{is}\;35\mathrm{and}\;\mathrm{over})+\beta_{4}I(j\;\mathrm{is}\;\mathrm{Rest}\;\mathrm{of}\;\mathrm{EU})+\beta_{5}I(j\;\mathrm{is}\;\mathrm{Other})$$

where *i* indexes age group “Up to 24 years”, “25–29”, “30–34” and “35 and over”, and *j* indexes maternal region of birth UK, Rest of EU and Other regions. *y*_*ij*_ is the number of births to an HCV seropositive woman, *n*_*ij*_ is the number of births, *p*_*ij*_ is the probability that a birth is to an HCV seropositive woman and *I* is the usual indicator function with *I*(event) = 1 if the event is verified and 0 otherwise. The parameter *β*_0_ corresponds to the baseline age group of “Up to 24 years” and maternal region of birth as the UK.

Inference about the unknown parameters *β*_0_, *β*_1_, *β*_2_, *β*_3_, *β*_4_ and *β*_5_ was performed in the Bayesian framework using Stan https://mc-stan.org/ run using the rstan package [28] in R version 4.2.1 [29], which was used for all other computations. The figures and supplementary figures were produced using ggplot2 [29, 30].We used independent scaled and shifted *t*_7_ distributions, with location 0 and scale 5, as prior distributions for the model parameters: $\beta_{i}∼t_{7}(\mathrm{location}=0,\mathrm{scale}=5),i=0,1,\ldots ,5.$ This *t*-distribution has heavier tails than a normal distribution, with the prior probability that *β*_*i*_ takes values between -20 and 20 being 0.99. The number of degrees of freedom for the *t* distributions were chosen as a trade-off between having heavier tails than the normal model and avoiding distributions with undefined variances. Stan draws samples from the posterior distribution of *β*_0_, *β*_1_, *β*_2_, *β*_3_, *β*_4_ and *β*_5_, that is from the distribution of the parameters after seeing the data. These draws can be converted into samples from the posterior distribution of the probabilities *p*_*ij*_ using the general conversion formula p=exp(η)/{1+exp(η)}wherelog{p(1‐p)}=η.

Four Markov chains were used in Stan, each of which was run for 50,000 iterations including a burn-in of 25,000 iterations. No thinning was applied, meaning that the overall number of values drawn from the posterior distribution of *β*_0_, *β*_1_, *β*_2_, *β*_3_, *β*_4_ and *β*_5_ was 100,000. S1 Table provides a summary of the results from Stan. The effective sample size is high and the value of $\hat{R}$ (not reported here) was very near 1 for all parameters [31], indicating good numerical performance of Stan for sampling from the posterior distribution. This is confirmed by the fact that all four Markov chains provided by Stan gave similar probability density functions (S1 Fig). We then combined draws from the posterior distribution of the associated probabilities *p*_*ij*_ with data on the number of livebirths in each category (*i*,*j*) in each of the nine regions of England in the two years 2013 and 2018 to infer the number of livebirths to HCV seropositive women (expressed as an absolute number and number per 100,000 livebirths) in each region in these years.

S2 Table shows the results that we obtained from Stan using other prior distributions (*t*- and normal) for the model parameters. We can see that our inference about the model parameters is fairly robust to the choice of prior distribution. Generally, the more the prior concentrates probability density around zero, the more the posterior mean is shrunk towards zero.

Two other models that are special cases of the above full model were considered. One had terms depending only on maternal age group in the linear predictor $\beta_{0}+\beta_{1}I(i\;\mathrm{is}\;25‐29)+\beta_{2}I(i\;\mathrm{is}\;30‐34)+\beta_{3}I(i\;\mathrm{is}\;35\;\mathrm{and}\;\mathrm{over})$ (age group only), while the other had terms depending only on maternal RoB $\beta_{0}+\beta_{4}I(j\;\mathrm{is}\;\mathrm{Rest}\;\mathrm{of}\;\mathrm{EU})+\beta_{5}I(j\;\mathrm{is}\;\mathrm{Other})$ (RoB only). It is usual in the Bayesian framework to choose between models based on information criteria or related quantities. These Bayesian model choice quantities have similar interpretations to Akaike’s information criterion or AIC [32], which is used as a penalised ‘badness-of-fit’ statistic in frequentist statistical inference [33]. The deviance information criterion or DIC [34, 35] is a Bayesian version of AIC and has been used very successfully for model comparisons. Nowadays, WAIC [36], computed using the R package loo [37, 38], is considered to be a generally better, but more computationally expensive, alternative to DIC, as it provides a fully Bayesian approach for assessing the out-of-sample predictive performance of a model (for examples see [38, 39]). Smaller values of WAIC are usually preferred. We obtained the following values of WAIC for the models that we considered (approximate standard errors in brackets): full 44.6 (6.1), Age group only 65.7 (12.2) and RoB only 42.2 (5.2). The difference in WAIC between the full model and the RoB only model was 2.4 (3.4), meaning that these models have very similar predictive performance. We choose to use the full model in order to benefit as much as possible from working in the Bayesian framework by taking account of the uncertainty associated with maternal age group.

In order to validate our model further, we computed the posterior predictive distribution [40] of new *y*_*ij*_ values, $y_{ij}^{\left(new\right)}$ say. This was done by sampling, for each (*i*,*j*) combination, $y_{ij}^{\left(new\right)}$~Bin(*n*_*ij*_, *p*_*ij*_) for each of the 100,000 posterior draws of *p*_*ij*_ given the complete original data. This may provide an optimistic validation of our model. The resulting posterior predictive probability mass functions are compared with the observed data [41] in S3 Fig. For all combinations of age group and RoB there is considerable predictive support for the observed data, suggesting that our model provides sensible predictions.

## Results

Of the 24,599 samples included in these analyses, 25 were HCV seropositive. Table 1 shows the distribution of HCV seropositive samples and total livebirths by maternal age group and maternal RoB; just under half of births were to UK-born mothers. Births to women born outside of the EU accounted for 35% (8624/24,599) of the total and 52% (13/25) of those to HCV seropositive mothers.

**Table 1:** The number of livebirths to HCV-seropositive women *y*_*ij*_/the number of livebirths *n*_*ij*_ by maternal age group and maternal RoB; data from the 2012 North Thames survey.

	Maternal Region of Birth *j*			Total
Age Group *i*	UK	Rest of EU	Other	
Up to 24	0 / 2505	1 / 481	0 / 1117	1 / 4103
25 to 29	0 / 3223	5 / 991	3 / 2495	8 / 6709
30 to 34	1 / 4065	2 / 1110	5 / 2909	8 / 8084
35 and over	2 / 2947	1 / 653	5 / 2103	8 / 5703
Total	3 / 12740	9 / 3235	13 / 8624	25 / 24599

**Fig 1:**
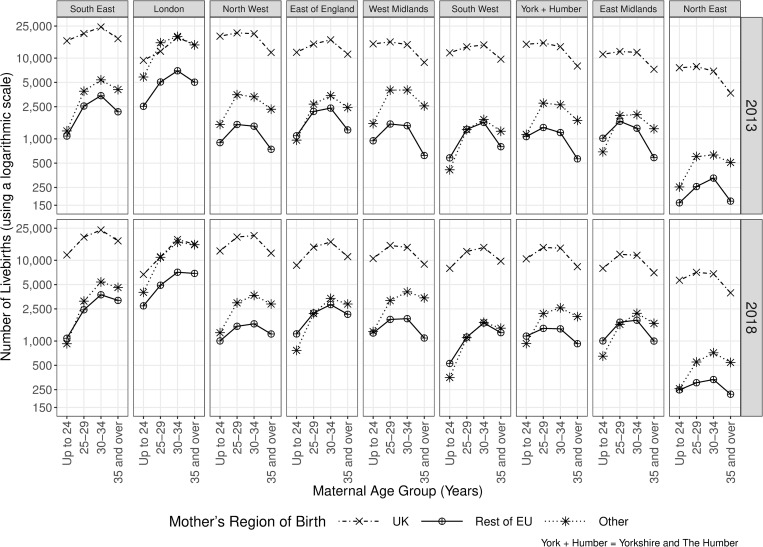
Number of livebirths in each of the nine regions of England in the years 2013 and 2018 by maternal age group and RoB. A logarithmic scale has been used for the number of livebirths.

Fig 1 shows the number of livebirths overall in each of the nine regions of England in 2013 and 2018 by maternal age group and RoB. A total of 664,517 livebirths were recorded in England in 2013, which fell to 625,651 in 2018. As shown in Fig 1, the majority of livebirths in all regions of England apart from London were to UK-born women, while in London the proportion of births to UK-born women was lower and similar to women born outside of the EU. Births among young mothers (less than 35 years old) decreased in all regions of England between 2013 and 2018; however, births among older mothers (35 years and older) increased or remained constant in all regions of England.

### Estimating the number of livebirths to HCV seropositive women in England in 2013 and 2018, nationally and by region

Our inferences about the model parameters allow us to estimate the probabilities *p*_*ij*_ in the logistic regression model, i.e. the probabilities of HCV seropositivity for different maternal age groups *i* and RoBs *j*. The posterior distributions of these probabilities are shown in S2 Fig, which confirms that the probability of maternal seropositivity is highest for the “35 and over” maternal age group. Similarly, the probability when the maternal RoB is the Rest of EU is higher than when it is Other regions, which in turn is higher than when it is UK.

**Fig 2:**
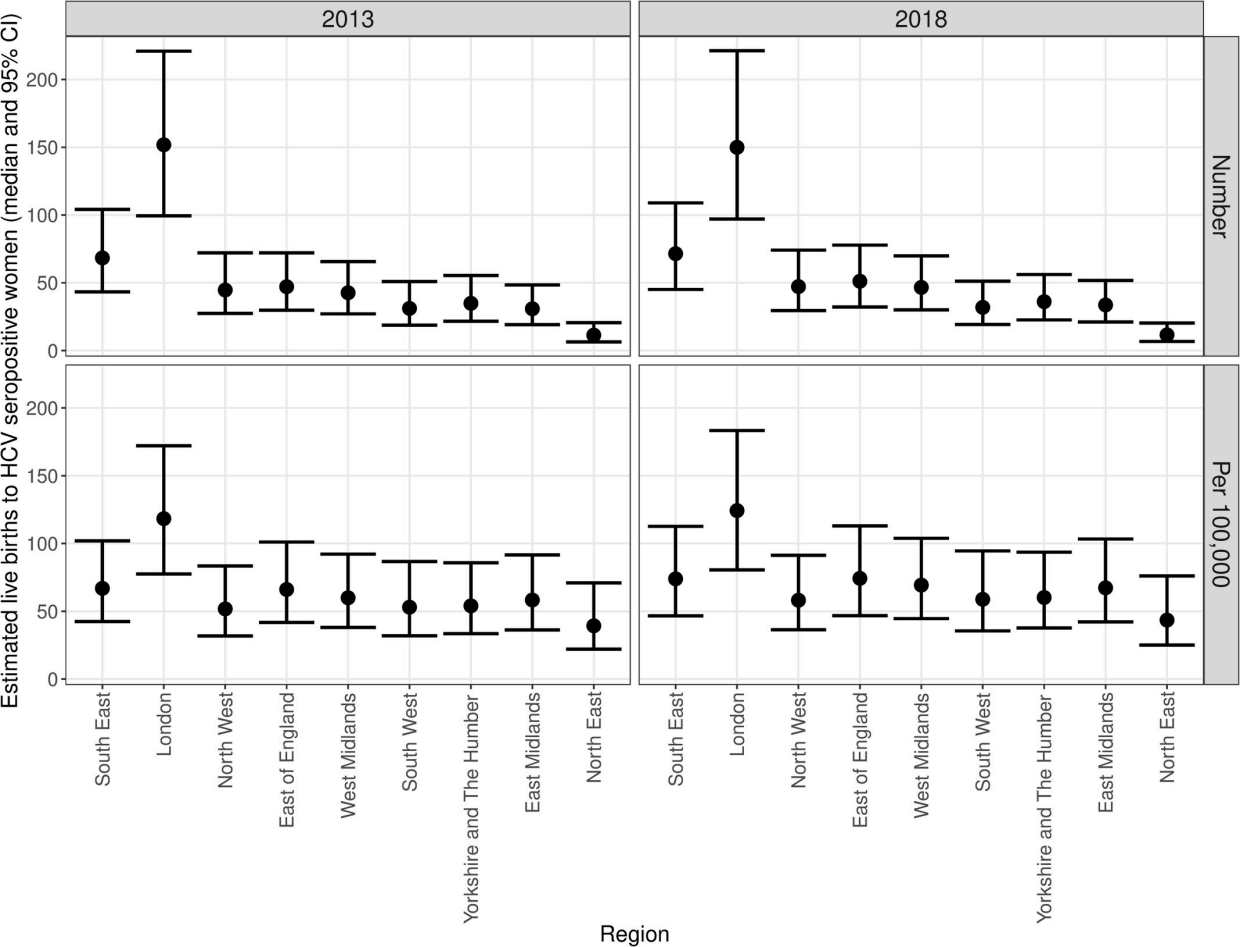
Estimated livebirths to HCV seropositive women expressed as absolute number and number per 100,000 livebirths, together with 95% credible intervals (CIs), for each region of England, for the years 2013 and 2018.

Table 2 and Fig 2 show the estimated number of livebirths to HCV seropositive women by region of England in 2013 and 2018. The regions with the highest estimated absolute number of livebirths to HCV seropositive women were London and the South East, the two regions with the highest numbers of livebirths overall; when expressed as a rate per 100,000, the estimated number of livebirths to HCV seropositive women remained highest in London (118.5 and 124.4 per 100,000 in 2013 and 2018 respectively), while the rate in the South East was similar to the East of England and West and East Midlands, at 67.0 and 74.0 per 100,000 in 2013 and 2018 respectively. Estimates were lowest for the North East, with 39.3 per 100,000 livebirths to HCV seropositive women in 2013 and 43.6 per 100,000 in 2018.

**Table 2:** Estimated number of livebirths to HCV seropositive women per 100,000 livebirths, together with 95% credible intervals.

	2013			2018		
		Credible interval			Credible interval	
Region	Value	Lower limit	Upper limit	Value	Lower limit	Upper limit
South East	67.0	42.5	102.1	74.0	46.8	112.5
London	118.5	77.7	171.8	124.4	80.8	183.1
North West	51.8	31.9	83.2	58.2	36.5	91.1
East of England	66.1	41.8	101.0	74.3	46.9	113
West Midlands	60.0	38.1	91.9	69.4	44.8	103.7
South West	53.1	32.0	86.5	58.9	35.6	94.5
Yorkshire and The Humber	54.0	33.5	85.5	60.2	37.9	93.3
East Midlands	58.4	36.2	91.5	67.3	42.4	103.2
North East	39.3	22.1	70.8	43.6	25.2	75.8

The estimated number of livebirths to HCV seropositive women for England as a whole was 464 with a 95% credible interval [CI] of 300–692 in 2013 and 481 (95%CI 310–716) in 2018. The corresponding numbers per 100,000 livebirths were 70.0 (95%CI 45.0–104.1) and 76.9 (95%CI 49.5–114.4).

## Discussion

We estimate that in England the number of livebirths to HCV seropositive women was 70.0 per 100,000 in 2013 and 76.9 per 100,000 in 2018, with the highest rates in London and lowest rates in the North East. We produced these estimates by applying maternal age and RoB-specific HCV seroprevalence data from North Thames in 2012 to the underlying demographic structure of the population in England delivering livebirths in 2013 and 2018. In this way we were able to account for changes in the demographic composition of the antenatal population over time, within the constraints of information available on HCV infection among different subgroups in the 2012 North Thames dataset (25 HCV seropositive samples among 24,599 livebirths).

The most common non-UK maternal countries of birth in England and Wales have remained fairly stable over these years, with the exception of Romania, which increased from a rank of ninth among most common non-UK maternal countries of birth in 2012 to third in 2018 [42]. The under-representation of women born in Romania in the 2012 data may therefore be a source of uncertainty in the 2018 estimates, as this suggests a change in the underlying composition of women in the ‘Rest of the EU’ category. In the 2012 study, Eastern Europe was the region with the highest HCV seroprevalence at 0.366%, although there were no Romanian women who were HCV seropositive [22]. Modelling estimates for Romania for 2019 placed the prevalence of viraemic HCV infections among women aged 15–49 years at 1.99%, and >2% in every age group ≥25 years [2], although prevalence of HCV infections may be different among migrant groups [43]. While the higher estimates for HCV seroprevalence among older age groups in our study is consistent with other studies [2], this differs from results of the 2012 analysis which showed, for women born in Eastern Europe, that HCV seropositivity was highest among younger women [22]. The difference is due to the re-categorisation of RoB data to match ONS datasets (UK, Rest of EU, Other), with Eastern European countries split into the Rest of EU and Other categories, thus diminishing the effect for younger women; other age and region-specific patterns may also have been undetected due to the required use of broad categories.

Although we were able to account for changes in underlying demographic structure, we were not able to model changes in age and RoB-specific probabilities of HCV infection over time, because the seroprevalence data were available for one time point only. This means that we had to assume that women of the same age and RoB delivering in 2012 and in 2018 had the same probability of being HCV seropositive, despite having been born six years apart. Overall, prevalence of HCV increases with age, reflecting the chronicity of the infection and ongoing exposures among risk groups, with global prevalence of viraemic infection estimated to be 0.25% in women aged 15–19 years increasing up to 1.21% in those aged 45–49 years [2]. However, changes in risk factors over time may also impact on risk of HCV infection specific to birth cohorts, for example, reductions in healthcare-associated acquisition risk linked with safer medical practices [44], or the increase in risk seen in the USA over the last decade linked with the opioid crisis [45, 46]. The 2012 North Thames data showed no HCV infections in UK-born women aged <31 years. Surveillance data in England on low risk populations stratified by RoB or ethnicity is limited, but data on new blood donors in 2018 showed substantially lower rates of HCV seropositivity among White-British donors (3.6 per 100,000) compared with “Other White background” (67.3 per 100,000) and South Asian ethnicities (66.6 per 100,000) [6].

We estimated that in 2018, 481 (95%CI 310–716) livebirths in England were to HCV seropositive women. A key question relates to the proportion of these babies likely to have been born to women with viraemic infection and therefore at risk of vertical transmission. Approximately 25% of individuals are estimated to spontaneously clear HCV following initial infection [47], while an estimated 38% of individuals in the UK with chronic HCV infection overall had been successfully treated by 2019 following the scale-up of DAAs [6]. The chance that pregnant women with HCV antibodies are treated before pregnancy or subsequently–and that their infants are diagnosed in the case of vertical transmission occurring–depends on the proportion whose HCV infection is already diagnosed under current policies. Two studies in London hospitals both reported that the majority of screen-positive pregnant women were undiagnosed prior to pregnancy: in one, assessing antenatal HCV screening between 2003–2013, 73% of viraemic women (who comprised 44% of seropositive women) were newly diagnosed through screening [12], and in a 2013 retrospective study of unlinked stored samples, two of the five seropositive women had a pre-pregnancy diagnosis [23]. Recent efforts to expand the reach of HCV testing services in the general population have included public campaigns to communicate risk factors for HCV infection and the benefits of testing, and targeted initiatives among people accessing community drug treatment services and socially excluded communities [6]. The extent to which these initiatives may have increased HCV diagnosis rates pre-pregnancy is unclear, and for recent migrants, the chance of a pre-pregnancy diagnosis will also depend on screening policies in countries of origin. Data on existing diagnosis rates specific to pregnant women are essential for informing analyses on the benefits and cost-effectiveness of any future antenatal screening programme.

Antenatal HCV testing is currently recommended for pregnant women at increased risk of HCV–including those newly diagnosed with HIV or HBV through antenatal screening, those with specific exposures or risk factors such as injecting drug use or homelessness, and those born or brought up in a country with a ≥2% prevalence of chronic HCV [48–50]. Risk-based screening of pregnant women in other countries follows differing protocols with respect to risk groups (e.g. some include women with an IDU history while others also extend to those with a sexual partner who inject drugs; other protocols include women from high-prevalence areas) [20]. A few studies have compared risk-based with universal screening, and have shown that women with a known risk factor, such as injecting drug use, may not be screened under current guidelines [20]. In England, although coverage of HCV testing among high-risk pregnant women is unknown, testing of migrant groups in primary care settings is variable. A recent survey of primary care professionals indicated that most HCV testing among existing migrant patients was done opportunistically rather than through systematic identification of high-risk patients, with only 17% of respondents stating that they offered universal opt-out HCV testing to newly registering migrant patients [51]. Specific barriers that migrants may face in accessing testing and subsequent linkage to care include language [52] (with a fifth of pregnant women screening positive for hepatitis B in England in 2014 having a less than basic level of English) [53], uncertainties around migrants’ entitlement to healthcare (which also exist among healthcare professionals) [51], and possible intersectional risk, particularly relating to IDU among migrants from Eastern Europe [54, 55]. Furthermore, the lack of identifiable risk factors for some women may fail to identify some infected women, as was seen more than 20 years ago with selective antenatal screening for HIV in the UK [56], resulting in missed opportunities for postpartum treatment or follow-up of the HCV-exposed child.

Residual neonatal DBS are a source of unbiased information on maternal infection due to the very high coverage of metabolic screening and the unconsented nature of residual DBS testing for unlinked anonymous studies [57]. However, around a fifth of the sample from the 2012 study had to be excluded from these analyses due to missing data on maternal age or RoB. This may have resulted in bias if maternal details were less likely to be available for particular groups, although the proportion with HCV antibodies in the excluded sample was broadly similar to those included. Importantly, our interpretation is limited by the lack of data on the proportion of HCV seropositive women who had chronic (viraemic) infections, which is key to understanding vertical transmission risk and the extent to which diagnosis and treatment initiatives in the wider population may be reaching women of reproductive age, particularly since 2012 and DAA roll-out. Our estimates of the number of seropositive women delivering livebirths assume that maternal age and RoB-specific probabilities of HCV antibodies are the same for other regions of England as for North Thames, which may not be accurate due to differences in risk factors by region (e.g. injecting drug use behaviours), or time since arrival in the UK. For example, prevalence of opioid dependence varies regionally, with the prevalence among 15–34 year old females in England in 2008–09 estimated at 0.41% (95% CI 0.30–0.59) in South East (with a similar prevalence in East of England), ranging to 0.87% (95% CI 0.64–1.27) in the North West, using a Bayesian modelling approach [58]. There have been declines in current injecting [59] and an increase in the average age of people who inject drugs overall in England over time [60]. The broad maternal RoB categories defined by the ONS that we used in our analyses (UK, Rest of EU, Other) may also have masked differences in the distribution of maternal countries of birth across regions of England or by year. Our findings should therefore be interpreted with some caution and the wide credible intervals that we report help with this.

This study presents methods that allow future national and regional estimates of maternal HCV seroprevalence to be produced and uncertainty associated with these estimates to be properly quantified. These methods could also be applied to other infections in pregnancy, for which national surveillance data are currently lacking. However, the assumptions and uncertainty around our estimates also highlight the need for further data on the epidemiology of HCV infection among pregnant women in England. Better region-specific information (which could be achieved through seroprevalence studies similar to that conducted in North Thames, repeated in other regions and time periods) could determine and improve the accuracy of our model-based approach. Crucially, however, there is a need for additional data on prevalence of maternal viraemic infections as well as diagnosis rates to inform interpretations. Taken together, these evidence gaps in the epidemiology of HCV in pregnant women constitute a barrier to the introduction of an antenatal screening programme which, paradoxically, need to be addressed via national surveillance in order to comprehensively assess the burden of maternal and vertically-acquired HCV infection in the UK.

In conclusion, the findings of this study suggest a low number of livebirths to HCV seropositive women among most sub-populations in England but with higher HCV seroprevalence among older and non-UK born mothers, and higher numbers of HCV-exposed pregnancies in London and the South East. Few previous studies have investigated HCV among pregnant women in England. These findings therefore complement and supplement existing research by providing national and regional estimates for the number of livebirths to HCV seropositive women in England and by properly quantifying the associated uncertainty.

## Supporting information

S1 Fig Posterior probability density functions for *β*_0_, *β*_1_, *β*_2_, *β*_3_, *β*_4_ and *β*_5_ for the four Markov chains used for sampling. (TIF)

S2 Fig The posterior distributions of the probability of HCV seropositivity *p*_*ij*_ in the logistic regression model shown as histograms. Posterior medians are shown using a vertical lines. Probability is displayed using a logarithmic scale so that small values can be seen more easily.
(TIF)

S3 FigPredictive probability mass functions for $y_{ij}^{new}$.The observed data *y*_*ij*_ are shown using a vertical line.(TIF)

S1 Table Summaries of the posterior distribution of *β*_0_, *β*_1_, *β*_2_, *β*_3_, *β*_4_ and *β*_5_. The posterior mean, standard deviation, 2.5 percentile, median, and 97.5 percentile are given, together with the approximate effective number of independent values drawn from the posterior distribution.
(DOCX)

S2 Table Posterior means, 95% credible intervals and interval widths for *β*_0_, *β*_1_, *β*_2_, *β*_3_, *β*_4_ and *β*_5_ for different prior distributions. The *t*-distributions have the same variance. Normal distributions are parameterized by their means and standard deviations.
(DOCX)

S1 Data (CSV)

S2 Data (R)
