# Supplementary figures and images for: Estimating the number of livebirths to Hepatitis C seropositive women in England in 2013 and 2018 using Bayesian modelling
Source: PLoS One. 2022 Nov 21;17(11):e0274389. doi: 10.1371/journal.pone.0274389 (PMC9678281; doi:10.1371/journal.pone.0274389)

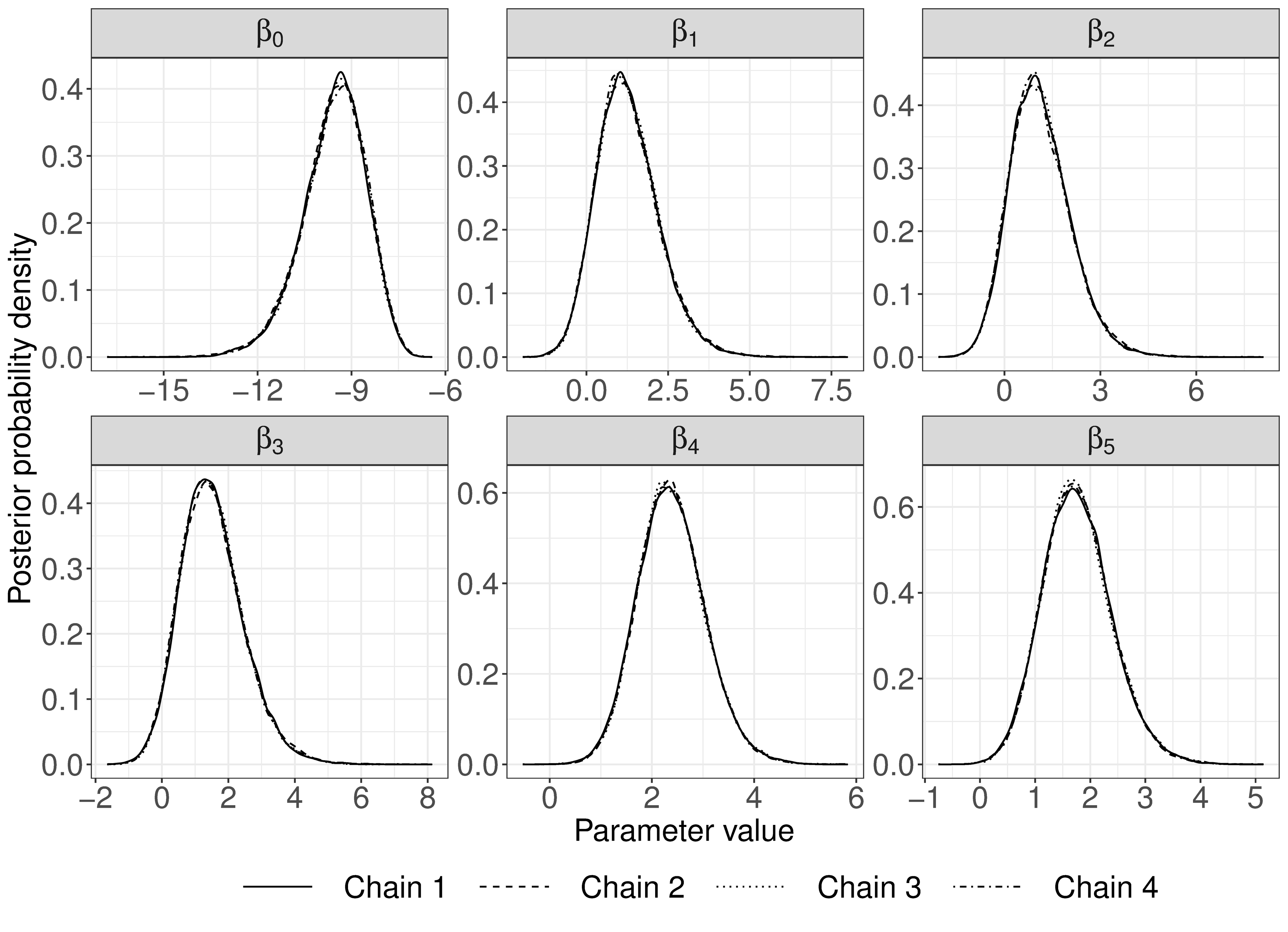

Supplement: S1 Fig — (TIF) [file pone.0274389.s001.tif]

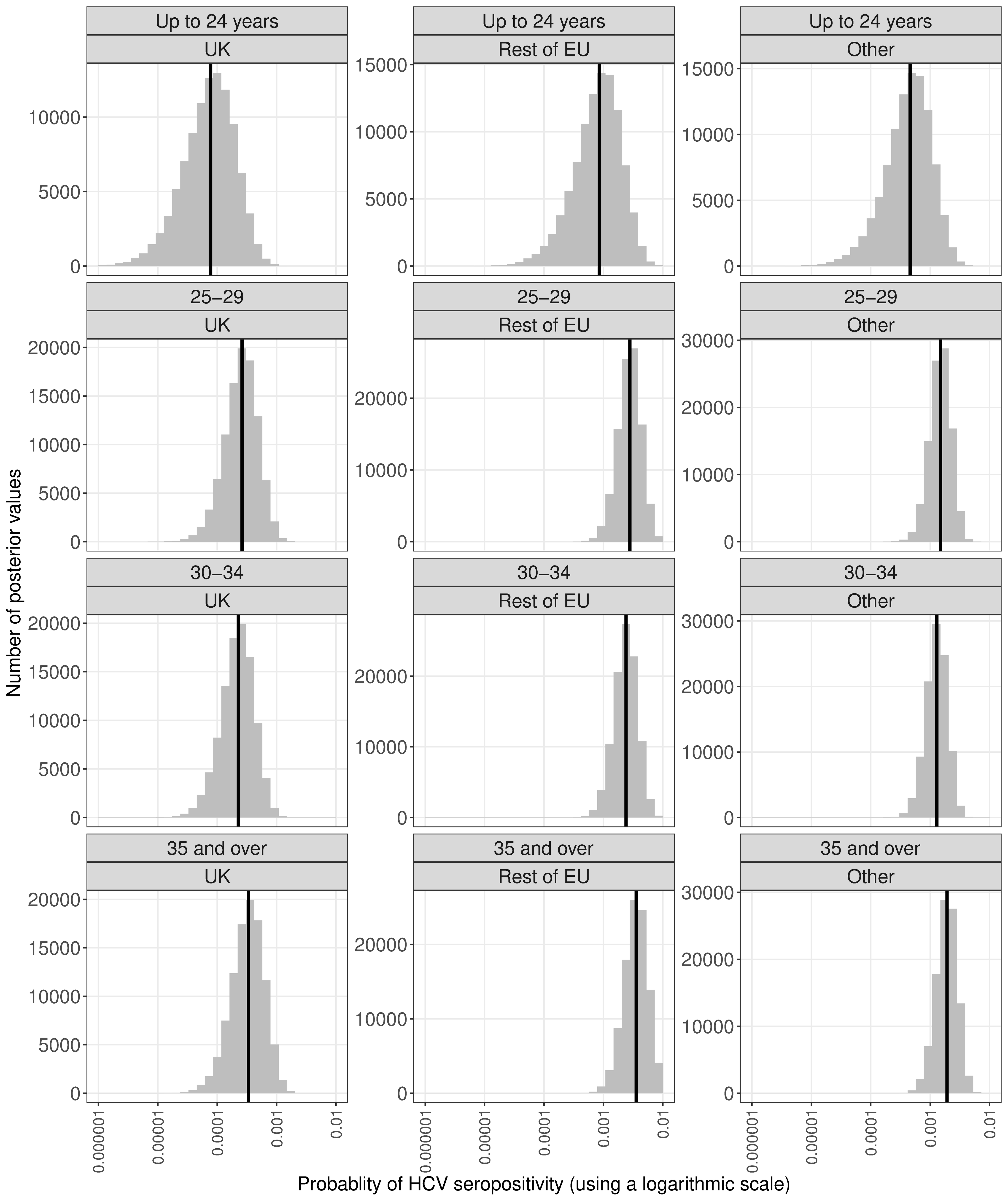

Supplement: S2 Fig — Posterior medians are shown using a vertical lines. Probability is displayed using a logarithmic scale so that small values can be seen more easily. (TIF) [file pone.0274389.s002.tif]

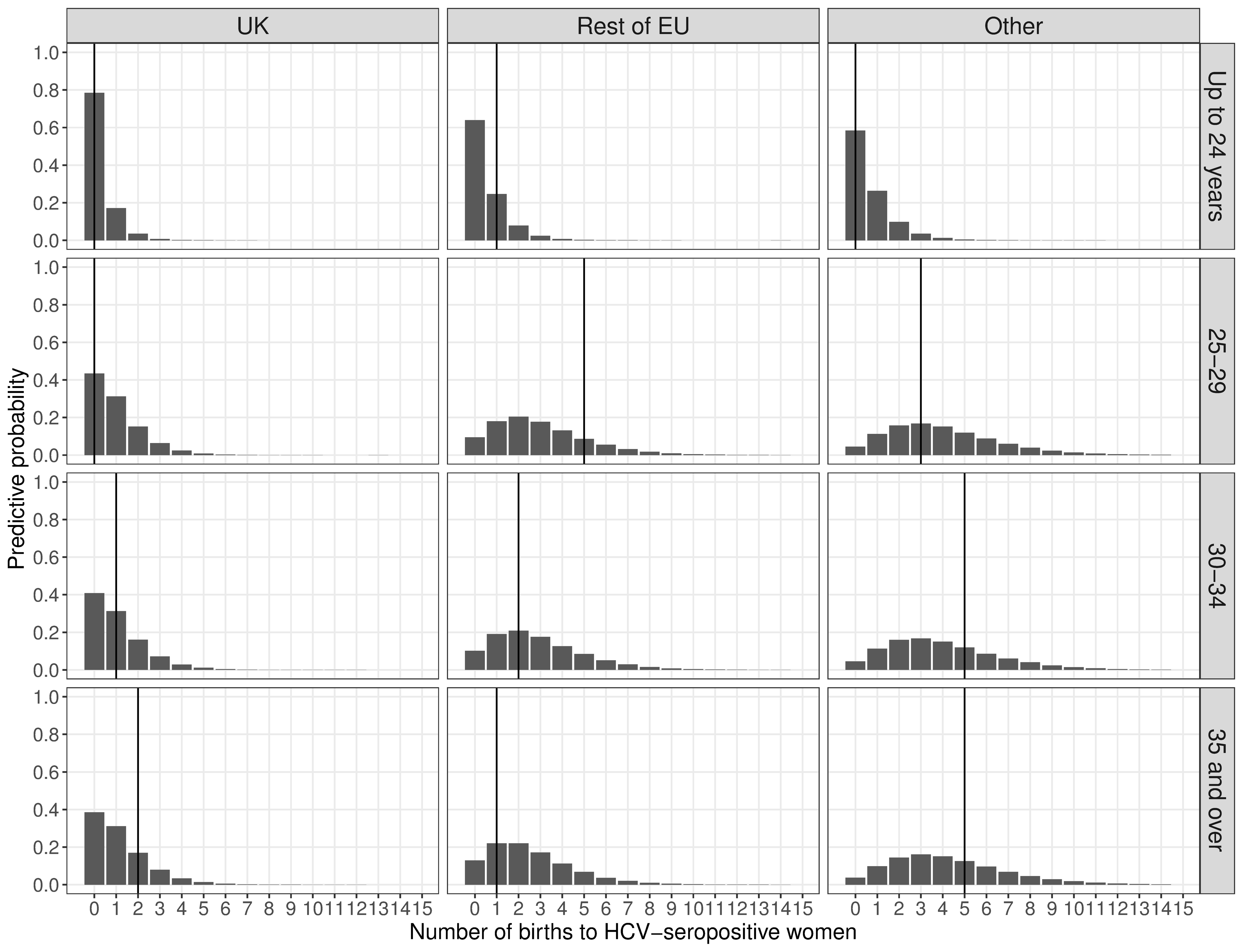

Supplement: S3 Fig — The observed data yij are shown using a vertical line. (TIF) [file pone.0274389.s003.tif]
