# Supplementary material for: Estimating the number of livebirths to Hepatitis C seropositive women in England in 2013 and 2018 using Bayesian modelling
Source: PLoS One. 2022 Nov 21;17(11):e0274389. doi: 10.1371/journal.pone.0274389 (PMC9678281; doi:10.1371/journal.pone.0274389)
Supplement: S1 Table — The posterior mean, standard deviation, 2.5 percentile, median, and 97.5 percentile are given, together with the approximate effective number of independent values drawn from the posterior distribution. (DOCX) [file pone.0274389.s004.docx]

Supplementary Table 1: Summaries of the posterior distribution of $\beta_{0}$, $\beta_{1}$, $\beta_{2}$, $\beta_{3}$, $\beta_{4}$ and $\beta_{5}$. The posterior mean, standard deviation, 2.5 percentile, median, and 97.5 percentile are given, together with the approximate effective number of independent values drawn from the posterior distribution.

| Parameter | Mean | Standard deviation | 2.5% | 50% | 97.5% | Effective number of independent values drawn from the posterior |
| --- | --- | --- | --- | --- | --- | --- |
| $\beta_{0}$ (<25 years; RoB: UK) | -9.57 | 1.02 | -11.83 | -9.47 | -7.84 | 23000 |
| $\beta_{1}$(25-29 years) | 1.28 | 0.96 | -0.36 | 1.20 | 3.39 | 24000 |
| $\beta_{2}$ (30-34 years) | 1.13 | 0.96 | -0.51 | 1.05 | 3.25 | 23000 |
| $\beta_{3}$ (35 years and over) | 1.52 | 0.96 | -0.12 | 1.44 | 3.64 | 24000 |
| $\beta_{4}$ (RoB: Rest of EU) | 2.38 | 0.66 | 1.17 | 2.35 | 3.78 | 33000 |
| $\beta_{5}$ (RoB: Other) | 1.77 | 0.63 | 0.63 | 1.73 | 3.11 | 34000 |
