# Supplementary material for: Estimating the number of livebirths to Hepatitis C seropositive women in England in 2013 and 2018 using Bayesian modelling
Source: PLoS One. 2022 Nov 21;17(11):e0274389. doi: 10.1371/journal.pone.0274389 (PMC9678281; doi:10.1371/journal.pone.0274389)
Supplement: S2 Table — The t-distributions have the same variance. Normal distributions are parameterized by their means and standard deviations. (DOCX) [file pone.0274389.s005.docx]

Supplementary Table 2: Posterior means, 95% credible intervals and interval widths for $\beta_{0}, \beta_{1}, \beta_{2}, \beta_{3} , \beta_{4}\text{and} \beta_{5}$ for different prior distributions. The *t*-distributions have the same variance. Normal distributions are parameterized by their means and standard deviations.

|  |  | | Prior Distribution | | | | | | |
| --- | --- | --- | --- | --- | --- | --- | --- | --- | --- |
| Parameter | | $t_{7}\left( 0,\text{ }5 \right)$ | | $t_{2.5}\left( 0,\text{ }\text{2.65} \right)$ | $t_{20}\left( 0,\text{ }5.61 \right)$ | N(0,5) | N(0,10) | N(0, 50) | N(0,100) |
| $\beta_{0}$ | | -9.57  (-11.83,-7.84)  3.99 | | -9.11  (-11.00,-7.61)  3.39 | -9.61  (-11.93,-7.84)  4.09 | -9.48  (-11.65,-7.79)  3.86 | -10.05  (-12.85,-8.03)  4.82 | -10.33  (-13.61,-8.15)  5.46 | -10.32  (-13.61,-8.14)  5.47 |
| $\beta_{1}$ | | 1.28  (-0.36,3.39)  3.75 | | 0.95  (-0.48,2.66)  3.14 | 1.31  (-0.37,3.47)  3.84 | 1.21  (-0.40,3.26)  3.66 | 1.66  (-0.23,4.34)  4.57 | 1.89  (-0.17,5.07)  5.24 | 1.89  (-0.17,5.07)  5.24 |
| $\beta_{2}$ | | 1.13  (-0.51,3.25)  3.76 | | 0.80  (-0.62,2.52)  3.14 | 1.16  (-0.51,3.31)  3.82 | 1.06  (-0.55,3.12)  3.67 | 1.51  (-0.40,4.20)  2.60 | 1.74  (-0.32,4.92)  5.24 | 1.74  (-0.32,4.93)  5.25 |
| $\beta_{3}$ | | 1.52  (-0.12,3.64)  3.76 | | 1.18  (-0.24,2.91)  3.15 | 1.55  (-0.13,3.71)  3.84 | 1.45  (-0.16,3.50)  3.66 | 1.91  (0.01,4.58)  4.57 | 2.14  (0.07,5.32)  5.25 | 2.14  (0.08,5.31)  5.23 |
| $\beta_{4}$ | | 2.38  (1.17,3.78)  2.61 | | 2.22  (1.07,3.52)  2.45 | 2.41  (1.18,3.82)  2.64 | 2.36  (1.15,3.73)  2.58 | 2.50  (1.24,3.98)  2.74 | 2.56  (1.27,4.09)  2.82 | 2.56  (1.27,4.07)  2.80 |
| $\beta_{5}$ | | 1.77  (0.63,3.11)  2.48 | | 1.62  (0.55,2.86)  2.31 | 1.78  (0.63,3.15)  2.52 | 1.75  (0.62,3.07)  2.45 | 1.88  (0.70,3.30)  2.60 | 1.94  (0.72,3.42)  2.70 | 1.93  (0.72,3.42)  2.70 |
